# The Therapeutic Potential of Cannabis in Counteracting Oxidative Stress and Inflammation

**DOI:** 10.3390/molecules26154551

**Published:** 2021-07-28

**Authors:** Michał Graczyk, Agata Anna Lewandowska, Tomasz Dzierżanowski

**Affiliations:** 1 Department of Palliative Care, Collegium Medicum in Bydgoszcz, Nicolaus Copernicus University, 87-100 Toruń, Poland; michal.graczyk@cm.umk.pl; 2 Collegium Medicum in Bydgoszcz, Nicolaus Copernicus University, 87-100 Toruń, Poland; aaw.lewandowska@gmail.com; 3 Laboratory of Palliative Medicine, Department of Social Medicine and Public Health, Medical University of Warsaw, 02-007 Warsaw, Poland

**Keywords:** cannabis, cannabinoids, inflammation, anti-inflammatory, antioxidative, immunology

## Abstract

Significant growth of interest in cannabis (*Cannabis sativa* L.), especially its natural anti-inflammatory and antioxidative properties, has been observed recently. This narrative review aimed to present the state of the art of research concerning the anti-inflammatory activity of all classes of cannabinoids published in the last five years. Multimodal properties of cannabinoids include their involvement in immunological processes, anti-inflammatory, and antioxidative effects. Cannabinoids and non-cannabinoid compounds of cannabis proved their anti-inflammatory effects in numerous animal models. The research in humans is missing, and the results are unconvincing. Although preclinical evidence suggests cannabinoids are of value in treating chronic inflammatory diseases, the clinical evidence is scarce, and further well-designed clinical trials are essential to determine the prospects for using cannabinoids in inflammatory conditions.

## 1. Introduction

Cannabis (*Cannabis sativa* L.) has been known and used since ancient times. It contains cannabinoids—A C21 terpene phenolic group of compounds, amino acids, fatty acids, steroids, along with secondary metabolites such as flavonoids, stilbenoids, terpenoids, alkaloids, lignans, and many others [1,2].

In recent years, significant growth of interest in the natural properties of its compounds has been observed, such as anti-inflammatory and antioxidative effects. They have been proved in numerous animal studies models and confirmed in clinical studies in patients suffering from inflammatory diseases, such as arthritis [3,4]. In terms of chemistry, the anti-inflammatory properties of cannabinoids can be related to an increase in glucocorticosteroid-like hormones production, which is used in anti-inflammatory therapy, and a decrease in prostaglandin synthesis, whose role in inflammatory conditions is commonly known [3].

This narrative review aims to present the state of the art of the research published in the last five years concerning the anti-inflammatory activity of all classes of cannabinoids, including (1) phytocannabinoids like Δ-9-tetrahydrocannabinol (THC) and cannabidiol (CBD); (2) their synthetic analogs like ajulemic acid (AJA; C_25_H_36_O_4_) and nabilone (C_24_H_36_O_3_); (3) endogenous cannabinoids like anandamide (N-arachidonoylethanolamine; AEA) and 2-arachidonoyl glycerol (2-AG); as well as (4) their derivatives like elmiric acids [5].

A likely mechanism of increased production of anti-inflammatory eicosanoids distinguishes cannabinoids from cyclooxygenase-2 inhibitors that suppress the synthesis of the pro-inflammatory eicosanoids [5]. Anti-inflammatory effects are also shown in non-cannabinoid compounds of cannabis—such as olivetol, cannflavins, and beta-caryophyllene (BCP)—a fragrant terpenoid known to be a full agonist of the CB2 receptor. CB2 is a G protein-coupled receptor, an important therapeutic target in many diseases [6,7]. Cannflavins A and B, on the other hand, appear to be cannabis specific plant flavonoids, known as flavones, which inhibit the production of prostaglandin E2 and the leukotrienes [8].

## 2. Endocannabinoid System (ECS) and Cannabinoids

**Figure 1:**
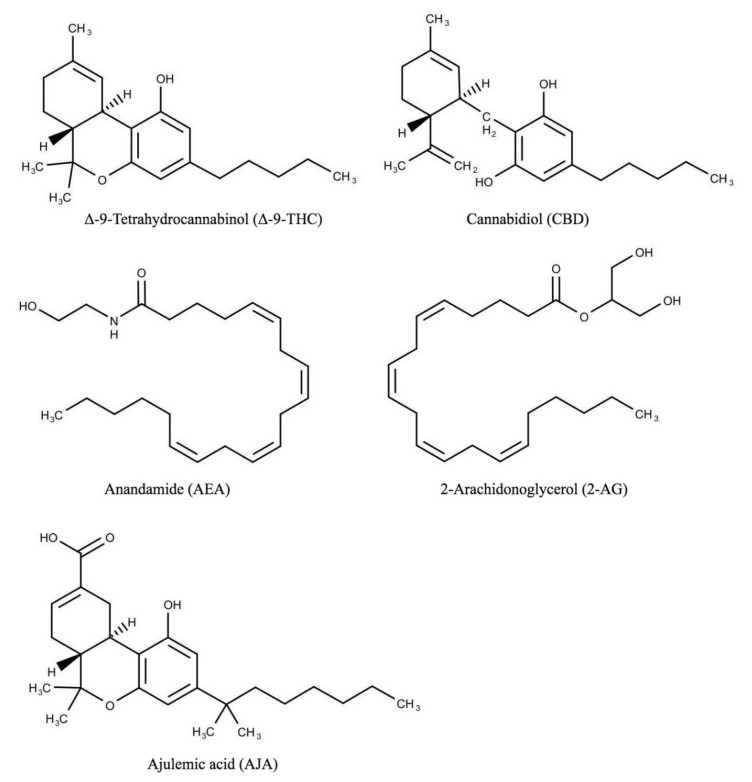
Structural formulae of main cannabinoids detailed in this review.

Cannabinoids can be classified as (1) endocannabinoids (AEA, 2-AG), (2) phytocannabinoids (THC, CBD), and (3) synthetic analogs (AJA)—Figure 1 [9]. Phytocannabinoids constitute more than 110 chemical compounds, while synthetic analogs are even more numerous [9]. The endocannabinoid system (ECS) consists of cannabinoid receptors CB1 and CB2, their endogenous lipid ligands—anandamide (AEA) and 2-arachidonoyl glycerol (2-AG), and the enzymes responsible for their biosynthesis (DAGLα, DAGLβ for 2-AG; NAPE-PLD for AEA) or degradation (fatty acid amide hydrolase—FAAH for AEA and monoacylglycerol lipase—MAGL for 2-AG) [10]. There are also alternative paths of endocannabinoid degradation, such as oxidation of AEA and 2-AG by cyclooxygenase, specific lipoxygenases, and P450 cytochrome [11]. AEA binds to central CB1 receptors and—to a lesser extent—peripheral CB2 receptors. 2-AG is a partial CB1 and CB2 agonist to which it binds with a comparable affinity [11]. Phytocannabinoids like Δ-9-tetrahydrocannabinol (THC) and cannabidiol (CBD) demonstrate a similar activity to anandamide and 2-AG.

THC is the main psychoactive cannabinoid due to its lipophilic structure capable of penetrating the blood–brain barrier and activating CB1 receptors widely expressed in the brain tissue [12]. CBD is the second of the two predominant phytocannabinoids of *Cannabis sativa* L. Compared to THC, CBD shows lower affinity to CB1 and CB2 receptors. Moreover, at low concentrations, it even demonstrates a slightly antagonistic effect and acts as a negative allosteric modulator of CB1—therefore indirectly changes the receptor’s potential to bind its orthosteric ligands, such as THC [13]. CBD is not psychoactive and shows numerous advantageous pharmacological effects, including anti-inflammatory and antioxidative properties. The chemistry and pharmacology of CBD have been thoroughly tested, together with various molecular targets, such as cannabinoid receptors and other compounds of ECS affected by CBD. Moreover, preclinical and clinical trials led to a better understanding of CBD’s therapeutical potential in many diseases, including those associated with oxidative stress [14].

Endocannabinoids seem to present affinity not only to CB1 and CB2 but also G protein-coupled receptors (GPR3, GPR6, GPR12, GPR18, GPR55, GPR119) [15], transient receptor potential vanilloid channels (TRPV1, TRPV2, TRPV3, TRPV4, TRPM8, and TRPA1), ligand-gated ion channels (5-HT3, glycine, nicotinic acetylcholine), and peroxisome proliferator-activated receptors (PPAR-α and PPAR-γ) [9,11,16].

CB1 receptors are located mainly in the central and peripheral nervous systems. They can also be found in the liver, pancreas, or gastrointestinal tract. CB2 receptors are located primarily in cells of the immunological system [17], but their presence has also been detected in the brain, heart, gastrointestinal tract, vessels, and endothelium [11]. The endocannabinoid system plays a crucial role in modulating immunological processes by decreasing major histocompatibility complex (MHC) class II on the surface of dendritic cells. It affects antigen presentation and inhibits peripheral T-cell activation in response to lipopolysaccharide and anti-CD3 antibodies [18]. Furthermore, cannabinoids can inhibit leukocyte proliferation, induce apoptosis of T lymphocytes and macrophages, and decrease the excretion of pro-inflammatory cytokines [19]. CB2 receptors are expressed in B lymphocytes, NK cells, monocytes, neutrophils, and leucocytes CD8 and CD4, making cannabinoids mitigate inflammatory response. Their immunomodulatory properties depend on the specific kind of the applied cannabinoid, dosage, the frequency of administration, and the cells they mediate [17]. Plasma and brain concentrations of CBD have been demonstrated to be strongly dose-dependent. The bioavailability of CBD and its half-life depend on the route of administration [20]. Cannabis-based medicines can be administered by smoking cannabis flowers, vaporizing oils or dry herbs and oral ingestion [21]. All the routes present different onset of effect, concentration stability, and potential health risks, which have to be thoroughly tested in order to determine the most precise pharmacokinetic profile, as well as minimize toxicity.

The main modes of action indicated by in vitro and in vivo pre-clinical studies are presented in Table 1.

## 3. Cannabinoids in the Inflammatory Bowel Diseases

The potential use of cannabinoids in inflammatory bowel diseases was a subject of research in recent years, not only on possible benefits associated with the anti-inflammatory effect but also the relief of the extraintestinal symptoms [22]. Although consecutive in vitro and in vivo research appeared promising, clinical trials are scarce [35]. Cannabinoids exert diverse effects on the digestive tract, regulating gastric hydrochloric acid secretion, motor activity, release and transport of ions, and visceral sensation [36].

CB1 and CB2 receptors are located in all layers of the bowel, including the myenteric and submucosal plexus and epithelium [17]. In vitro research confirmed the presence of CB1 and CB2 receptors in healthy human colon tissue, along with their reactivity to inflammation and epithelial injury [37]. Apart from CB1 and CB2 receptors, GPR55 and PPAR-α receptors have also been detected in the canine alimentary tract [38].

Increased expression of CB1 receptors in inflamed mucosa has been shown both in Crohn’s disease and ulcerative colitis patients [18]. Moreover, CB2 receptor agonists inhibit the release of interleukin-8 induced by tumor necrosis factor α (TNF-α) in human colon epithelial cells, which can significantly affect the immunological homeostasis of the intestine [31]. There is evidence for changes in expression and levels of endocannabinoids based on biopsies obtained from patients suffering from gastrointestinal diseases, such as diverticulitis, coeliac disease, irritable bowel syndrome, inflammatory bowel diseases, and colon cancer [11].

Mice subjected to genetic ablation of CB1 receptors were more susceptible to inflammatory injuries, which provides evidence of the protective role of CB1 receptors in case of inflammation. In mice with a genetic deletion of FAAH—the primary enzyme degrading anandamide—caused an increase in the concentration of anandamide in tissues. Mice with FAAH deficiency presented significant protection from dinitrobenzenesulfonic acid-induced colonic inflammation compared to the control group [39]. In another study, trinitrobenzenesulfonic acid was used in order to induce colonic inflammation in mice. The group deprived not only of CB1 receptors but also CB2, or both receptors experienced intensified inflammation compared to the control group [40]. The endocannabinoid system is stimulated in the process of intestinal inflammation in humans and animals. The activation of CB1 receptors can limit inflammation by regulating motor neuron activity and induction of healing epithelial injuries, which has been demonstrated in human colonic tissue research in vitro [41].

Another study tested the influence of 2-AG on the inflammatory response. Trinitrobenzenesulfonic acid was used to induce colitis in mice, followed by administering the MAGL inhibitor responsible for 2-AG degradation. Achieved results included a macroscopic and histological decrease in inflammatory changes, together with a reduction of expression of pro-inflammatory cytokines [42]. A similar study based on increasing levels of 2-AG in mice after administering the MAGL inhibitor showed a significant decrease in inflammation in the colon associated with mitigating systemic reaction, which further confirmed the protective role of 2-AG [43].

Apart from the classical path, cannabinoids, both natural and synthetic, seem to show affinity to G protein-coupled receptor 55 (GPR55) [23]. The involvement of GPR55 in the development of neuropathic and inflammatory pain by modulation of releasing pro-inflammatory cytokines has been proven, and the effects seem to be contradictory to those caused by cannabinoid receptors [15]. A pharmacological blockade achieved by administering a specific inhibitor in mice with experimentally induced colitis led to reducing lymphocyte and macrophage recruitment. Additionally, it caused a decrease in COX-2 expression, an inflammatory marker, as well as pro-inflammatory cytokines, such as IL-1β and TNF-α [15]. A possible protective capacity of cannabinoids against carcinogenesis in the colon is likely due to the inhibition of a release of pro-inflammatory cytokines, such as IFNγ, TNF-α, IL-17A, and IL-22 [25]. Further research is necessary, however.

The influence of cannabinoids, such as THC or CBD, in treating inflammatory bowel diseases in humans seems promising, although clinical trials evaluating their therapeutic potential are very limited and based only on small research groups, mostly Crohn’s disease and ulcerative colitis.

One hundred ulcerative colitis and 191 Crohn’s disease patients were enrolled in an observational study [44]. Cannabis appeared commonly used by patients with inflammatory bowel diseases to achieve symptom relief and improve the quality of life. A similar observational study included 313 patients with an inflammatory bowel disease who were using cannabis, mostly inhaled. Patients reported relief of abdominal pain, abdominal contractions, joint pain, and diarrhea. On the other hand, using cannabis was associated with adverse side effects and a higher risk of surgical intervention in patients with Crohn’s disease [45].

Another prospective study included 13 patients with chronic inflammatory bowel diseases [46]. After three months of treatment with cannabis, patients reported improved quality of life, clinical disease activity, and an increased body mass index. In a double-blinded placebo-controlled randomized clinical trial with 21 patients with active Crohn’s disease enrolled, the effects of eight-week-long treatment with cannabis cigarettes containing 115 mg of THC were assessed. The experimental group reported a higher response rate to the treatment and clinical remission. Side effects were evaluated as mild [41]. In another randomized clinical trial, a group of 19 active Crohn’s disease patients using cannabis oil consisting of 5% CBD was compared with the placebo group [41]. However, the study did not show any clinically relevant differences in remissions. In another trial, 50 patients with active Crohn’s disease were randomized to the experimental group, receiving cannabis oil consisting of 15% CBD and 4% THC, and the placebo group. After eight weeks, there was no clinical remission in the cannabis oil group, but patients reported a higher quality of life, and the Crohn’s disease activity index was observed to be lower than in the placebo group [41].

The existing research showing therapeutic benefits of using cannabinoids in inflammatory bowel diseases does not allow to draw unambiguous conclusions. Nonetheless, it may constitute a solid basis for further clinical trials (Table 2).

## 4. Cannabinoids in Inflammatory Skin Diseases

In human skin, cannabinoid receptors CB1 and CB2 are located in keratinocytes, hair follicles, sebaceous glands, sensory neurons, cells of the immune system, and fibroblasts [9,47,48]. FAAH and MAGL were also identified in the skin and its appendages, suggesting that it actively regulates its metabolic processes [9]. The ECS seems to have an impact on various dermal effects. Cannabinoids inhibit the proliferation and differentiation of epidermis keratinocytes and conduce to their apoptosis [28,47,49,50]. Additionally, stimulating CB2 causes the release of opioid peptides, which leads to analgesic effects [51]. Cannabinoids also participate in the modulation of the development and function of hair follicles and sebaceous glands. They significantly affect neuro-immuno-endocrine regulation of skin functioning and preserving its homeostasis [52,53]. It seems crucial that the ECS takes part in the coordination of the inflammatory response in the skin [9,47,49,52,54,55]. Functioning of the complex immunological protective barrier relies on the cooperation of different immune cells—such as macrophages, mast cells, T lymphocytes, dendritic cells, and Langerhans cells—together with keratinocytes, fibroblasts, melanocytes, and other cells present in the skin. The cooperation is complemented by receptors and pro- and anti-inflammatory cytokines and chemokines [49]. Dysfunction of this system can be observed in many diseases, such as atopic dermatitis, psoriasis, scleroderma, acne, dermatomyositis, keratin and hair growth disorders, carcinogenesis, together with symptoms such as pruritus, which shows potential for the future use of cannabinoids in the therapy of these disorders [9,28,49,52,56,57,58,59,60].

CB2 receptor agonists were studied for their potential in reducing inflammation and wound healing in mouse skin [32]. CB2 receptor activation led to reduced infiltration of neutrophils and macrophages, increased keratinocyte proliferation, and faster wound healing. Moreover, the expression of monocyte chemoattractant protein-1 (MCP-1), stromal cell-derived factor 1 (SDF-1), IL-6, IL-1β, TNF-α, transforming growth factor-beta 1 (TGF-β1), and vascular endothelial growth factor (VEGF) were also decreased. CB2 agonists lead to a significant decrease in pro-inflammatory M1 macrophages and a slight increase in anti-inflammatory M2 macrophages. Analogously, there was observed a decrease in gene expression, levels of proteins associated with M1 macrophages, and a release of cytokines (IL-6, IL-12, CD86, inducible nitric oxide synthase—iNOS), along with an increase in levels of cytokines associated with M2 macrophages (IL-4, IL-10, CD206, and arginase-1) [32]. In another study, authors demonstrated a decrease in pro-inflammatory factors, such as IL-6 and MCP-1, an increase in an anti-inflammatory factor—TGF-β, and faster wound healing after using a CB2 agonist [61]. Similarly, beta-caryophyllene, a CB2 receptor agonist, caused skin wound epithelialization by increasing the proliferation and migration of keratinocytes in mice [62].

It has been detected that levels of anandamide and 2-AG increase in mouse skin after experimentally inducing allergic contact dermatitis [63]. Moreover, mice deprived of both cannabinoid receptors show a more severe inflammatory reaction. Using CB1 and CB2 receptor agonists resulted in the attenuation of the inflammatory response, while the antagonists-exacerbation [63]. The influence of CB2 receptor agonists on artificially induced dermatitis in mice improved edema and skin lesions [64]. Presented research unambiguously points out that CB2 receptors, as a part of the ECS, impact the inflammatory reaction in the skin. Furthermore, the local application of CB1 agonists shows positive effects in mitigating inflammatory symptoms in the skin in an animal model [59].

Cannabinoids limit the activation and differentiation of mast cells by CB1 receptor stimulation, which can be beneficial in treating chronic inflammatory skin disorders [28,29]. Additionally, it has been proved that CB1 receptor activation by AEA inhibits the release of pro-inflammatory cytokines, such as IL-12, IL-23, and INF-γ by T lymphocytes in vitro. The effects can be inverted by inhibiting the CB1 receptor [30]. The demonstrated anti-inflammatory activity of AEA is especially important as CBD directly inhibits the reuptake of AEA, and therefore may alternate the inflammatory response [16].

Another study revealed that experimentally induced skin wounds in mice resulted in increased expression of FAAH, CB1, CB2, and PPAR-α, present in the epidermis and dermal appendages. Moreover, FAAH inhibitors turned out to effectively attenuate dermatitis in mice, thereby presenting the potential in treating inflammatory skin disorders [48]. Beagles allergic to dust mites were tested for the influence of endocannabinoid membrane transporter inhibitor on pruritus and dermatitis. Increasing levels of endocannabinoids improved atopic dermatitis and caused pruritus to alleviate after dust mite provocation in the experimental group [65].

Research undeniably shows active participation of CB1 and CB2 receptors in inflammatory skin disorders, although the functions of cannabinoids are not limited to the classic receptor path [26,47,49,54,66]. Topical application of THC effectively improves allergic contact dermatitis both in wild-type mice and the CB1 and CB2 receptor-deficient groups [26]. The study indicated that the properties of THC inhibiting the production of IFN-γ by T lymphocytes and decreasing the release of pro-inflammatory chemokines and cytokines induced by IFN-γ are independent of cannabinoid receptors [26]. This finding constitutes an essential step in identifying alternative functions of cannabinoids.

Transient receptor potential vanilloid-1 (TRPV1), highly permeable to calcium ions, affects cell proliferation, apoptosis, cytokine release, and T lymphocyte activation [67]. The activity of palmitoylethanolamide (PEA) and AEA in the epidermis includes, among others, mediation through TRPV1 [47,66]. Mice with TRPV1 deficiency show increased macrophages and expression of pro-inflammatory cytokines, such as TNF- α, IL-1β, and IL-6, compared to the control group [68]. Effects of genetic ablation of TRPV1 in mice include systemic inflammatory reaction and, reversely, a reduction of inflammation after administering TRPV1 agonists. It suggests the protective and anti-inflammatory role of TRPV1. The reports challenge the belief about pro-inflammatory properties of TRPV1 [67]. Moreover, certain cannabinoids can influence other channels of the TRP family, such as transient receptor potential ankyrin 1 (TRPA1), TRPV2, and TRPV3 [9,47], but the specific effects are not yet determined.

Palmitoylethanolamide (PEA) is an anti-inflammatory mediator, which intensifies the activation of cannabinoid receptors by AEA, activates TRPV1 and peroxisome proliferator-activated receptor α (PPAR-α) [69], which is the key regulator of inflammation and pain [70]. PPAR-α controls keratinocyte differentiation, wound healing and attenuates inflammation in skin disorders [71]. In mice, reduced itching and inflammation proved the therapeutic potential of PEA and was afterward reversed by using both PPAR-α and CB2 antagonists. The experiment showed the participation of both receptors in the anti-inflammatory effect [72]. In another study, contact allergic dermatitis in mice induced by dinitrofluorobenzene increased AEA, PEA, TRPV1, PPAR-α, and enzymes responsible for PEA biosynthesis [69].

Benign and malignant skin tumors also show expression of CB1 and CB2 receptors. It raises interest in the potential anticancer properties of cannabinoids [9]. In vivo studies proved the anti-inflammatory properties of cannabinoids and showed their potential in inhibiting carcinogenesis induced by 12-O-tetradecanoylphorbol-13-acetate (TPA) [73]. Ajulemic acid (AJA) is a synthetic cannabinoid preferentially binding to CB2 receptors, which might play a key role in inhibiting tumor progression through impeding IL-1β release responsible for inflammation in the microenvironment of the tumor [34]. THC seems to have the potential to inhibit the growth of melanocarcinoma [74]. However, the necessity of further research is substantiated by the results of a study that compared the influence of prolonged UVB irradiation [75]. Short exposure to UVB showed a much higher incidence of inflammation with an increased TNF-α in wild-type mice than CB1/2-deficient mice. The data suggest that UV irradiation directly activates CB1 and CB2 receptors, induces pro-inflammatory cascade, and, after prolonged exposure, leads to carcinogenesis [75]. Authors of the study recommend caution given the unclear and sometimes contradictory immunomodulatory effects of cannabinoids and draw attention to the necessity of further research [74].

Despite the limited number, clinical trials presented a significant decrease in pruritus after cannabinoid treatment in some dermatological disorders, such as atopic dermatitis, psoriasis, contact eczema, allergic contact dermatitis, and systemic conditions like uremic or cholestatic pruritus [56,57,58].

## 5. Cannabinoids in the Inflammatory Respiratory System Diseases

Immunological effects of cannabinoids imply the possibility of their therapeutic usage in respiratory tract disorders associated with inflammation [76]. The ability to dilate bronchi and the anti-inflammatory effect suggest the potential of cannabinoids in treating inflammatory and obstructive airway diseases. Preclinical research revealed the beneficial effects of the administration of CB1 agonists to alleviate experimentally induced contractions in the airways [77,78,79]. There are also reports of CB2 receptor involvement in counteracting bronchi contractions [80]. Apart from the anti-inflammatory and spasmolytic effects, in guinea pigs, the activation of CB2 receptors also inhibited cough reflex [81]. However, the significance of the mentioned properties is still unknown [82]. In another study, CB1 receptors appeared involved in the airway dilatation [83] and CB2 receptors in inhibition of activation of mast cells and eosinophils [79]. The potentially beneficial effects of CB1 and CB2 receptor activation in the airways were observed in guinea pigs with the induced asthma-like reaction after administration of a non-specific agonist [83]. In the experimental group, cough, suffocation, and airway obturation improved, along with decreased eosinophil infiltration, mast cell activation, free radicals release, and levels of TNF-α and prostaglandin D2 levels (PGD-2) compared to the control group [83].

Another study tested the influence of the MAGL inhibitor on inflammation in acute lung injury induced by lipopolysaccharide in mice. 2-AG decreased leucocyte migration to the lungs, vascular permeability, and levels of pro-inflammatory cytokines, such as TNF-α and IL-6 [84]. Decreased expression of CB2 receptors seems to be one of the mechanisms sustaining chronic inflammation in chronic obstructive pulmonary disease and is accompanied by increased pro-inflammatory cytokines, such as TNF-α, fibroblast growth factor β (bFGF), and TGF-β [85].

Another study confirmed the involvement of CB2 receptors in the course of respiratory syncytial virus (RSV) infection in mice [86]. Activation of those receptors significantly limited immune cell infiltration in the lungs of the infected animals and decreased neutrophils and monocytes counts in the broncho-alveolar fluid. The effects were accompanied by a decrease in IFN-γ and macrophage inflammatory protein-1α (MIP-1α) and an increase in IL-10 [86]. Administering a CB1 agonist also attenuated the inflammation in mice with RSV infection [87]. Overall, research shows the potential of anti-inflammatory properties of cannabinoids in treating RSV infection.

Reports suggest possible benefits of using CB2 agonists in limiting inflammatory response in patients infected with SARS-CoV-2, which arose from the abilities of the cannabinoid receptors to decrease the production of pro-inflammatory cytokines and immune cell proliferation [33]. One hypothesis suggests that CBD, as a non-psychotropic phytocannabinoid, can limit the severity and progression of the coronavirus disease 2019 (COVID-19) for several reasons. Firstly, high-cannabidiol extracts (from *Cannabis sativa* L.) are able to downregulate the expression of two key receptors for SARS-CoV2 in several models of human epithelia [24]. Secondly, CBD exerts a wide range of immunomodulatory and anti-inflammatory effects and can mitigate uncontrolled cytokine production responsible for acute lung injury [24]. Thirdly, being a PPAR-γ agonist, it displays a direct antiviral activity, and finally, PPAR-γ agonists are regulators of fibroblast/myofibroblast activation and can inhibit the development of pulmonary fibrosis, thus ameliorating lung function in recovered patients [24].

Special attention must be paid to the reports showing that CB2 receptors significantly contribute to allergic diseases associated with excessive eosinophil activity, such as bronchial asthma [88,89]. Pathological activation of eosinophils leads to the release of pro-inflammatory cytokines and effects such as excessive mucus production and tissue remodeling in the airways [89]. CB2 receptors are intensively expressed in eosinophils and monocytes [76], especially in patients with active allergy symptoms [89]. CB2 agonist in mice with experimentally induced inflammation of the airways induced the intensified migration of eosinophils to the respiratory tract and the exacerbation of airway hyperreactivity. The changes were absent in mice with eosinophil deficiency, suggesting that eosinophils are the main effector of the administered CB2 agonist. Effects at the cellular level have shown eosinophil shape change, increased chemotaxis, adhesion, and levels of reactive oxygen species. There was no eosinophil degranulation [89].

In another study, increased numbers of NK cells in the respiratory tract of CB2 receptor-deficient mice were detected [88]. NK cells, significant in bronchial asthma development, show high expression of CB2 receptors. In order to determine the meaning of the finding, the authors experimentally induced airway inflammation in mice by inhalation of dust mites. The wild-type group experienced a more severe inflammatory reaction and an increased mucus production, along with increased eosinophils, lymphocytes, and eosinophil peroxidase. In comparison, in the group with CB2 deficiency, the allergic reaction was significantly attenuated, and parameters such as eosinophils, T lymphocytes, and pro-inflammatory cytokines, like IFN-γ, were respectively lower. The results of the study indicate that CB2 receptor-deficient mice are resistant to allergen-induced inflammatory reactions. Administering a CB2 agonist in the next part of the experiment resulted in significant exacerbation of the eosinophil inflammation in the airways of wild-type mice [88]. Thus, CB2 receptor signaling in NK cells seems to be important in treating allergic diseases of the respiratory tract.

Administering CBD in experimentally induced allergic asthma in mice limited the inflammatory process and airway remodeling based on decreased collagen fibers and inflammatory markers [90]. However, the results of the preclinical studies are inconsistent and insufficient to draw unambiguous conclusions.

Anti-inflammatory properties of CBD have also been documented in inflammation artificially induced by lipopolysaccharide in mice. Lung function improvement and a decrease in leucocyte migration and lower levels of inflammatory markers—such as TNF, IL-6, MCP-1, or MIP-2—have been reported [91]. Furthermore, it has been proved that CBD can reduce cytokine storms and has a protective effect on lung tissue of mice with artificially induced acute respiratory distress syndrome (ARDS) [92]. There is a connection between CBD administration and the regulation of apelin—a peptide showing the protective effect on lung tissue [93].

Administration of THC also significantly attenuated the induced inflammation and the immunological response in the airways in mice [94]. The effects were observed even with simultaneous blockade or deficiency of CB1 and CB2 receptors, which points to equally relevant involvement of alternative paths of cannabinoids in mitigation of the inflammatory response [94]. In another study, authors proved the influence of THC in reducing the proliferation of immune cells and inhibiting the production of pro-inflammatory cytokines—such as IFN-γ, IL-1β, IL-2, or TNF-α—in mice with airway inflammation induced by Staphylococcal enterotoxin B (SEB) [95]. In a similar study, treating experimental groups with THC led to decreased alveolar macrophages, neutrophils, lymphocytes CD4+, CD8+, NK, and NKT cells, despite the administered toxin [96]. Furthermore, THC induces apoptosis of mononuclear cells infiltrating lungs and modifies the metabolism of T lymphocytes, which has been established based on reduction of cellular respiration in groups treated with THC, compared to the control group [96]. Moreover, THC reduces the mortality of mice with ARDS induced by SEB [97]. Research shows the potential of THC, as an anti-inflammatory agent, in treating cytokine storm and ARDS in patients suffering from COVID-19 [98].

On the other hand, adverse effects of cannabinoids on respiratory tract function in several pathological conditions have also been documented [99,100]. Excessive stimulation of CB1 receptors might be associated with lung injury, inflammation, and fibrosis, as well as increased pro-inflammatory cytokines and cyclooxygenase in the lungs [99].

Cannabinoids unquestionably affect immunomodulatory processes in the respiratory tract, showing significant anti-inflammatory properties. However, activation of CB1 and CB2 receptors often presents contrary effects in pathological conditions, which does not allow to determine their exact specific role in the airways [99].

## 6. Cannabinoids in the Neurological Disorders

The expression of CB1 receptors is remarkably high in the central nervous system (CNS), especially in the olfactory bulb, hippocampus, basal ganglia, and cerebellum [101]. Although CB2 receptors are mainly located in immune cells, their presence has also been detected in the CNS, making them an attractive potential target in counteracting inflammation in the nervous system [101]. A significant increase in expression of CB2 receptors in inflamed microglial cells associated with changes in levels of pro- and anti-inflammatory cytokines suggests the neuroprotective effects of the ECS [102]. Moreover, activation of CB2 receptors reduces recruitment and adhesion of neutrophils to the brain’s epithelium [103]. Changes in the expression of CB1 receptors also seem to be of importance [101]. CB1 receptors are involved in protecting against cell apoptosis by reducing excessive calcium release, and as a result—excitotoxicity [104]. CB1 receptors protected GABAergic neurons by reducing excitatory currents in experimentally induced autoimmune encephalomyelitis in mice [105].

The properties of cannabinoids to counteract oxidative stress in mitochondria seem to be meaningful [106]. Some reports present neuroprotective and anti-inflammatory activity of cannabinoids in the CNS by reducing cytotoxic elements in microglial cells, such as reactive oxygen species, pro-inflammatory cytokines, or nitric oxide [107,108]. Therefore, the role of the ECS is of significance in the case of neurodegenerative diseases, such as Alzheimer’s, Parkinson’s, Huntington’s diseases, or multiple sclerosis (MS) [101,105,106,107]. Literature offers evidence for the beneficial effects of CBD or THC treatment in nervous system diseases [109,110,111,112,113]. The complexity of the ECS functions in the CNS exceeds the scope of this article that focuses exclusively on the anti-inflammatory effects based on most common disorders.

Analysis of dopamine deficits in animal models led to the conclusion that in Parkinson’s disease, neuroprotective properties refer rather to CB2, not CB1 receptors [114]. CB2 receptor mediation in the treatment of neurodegenerative disorders could be beneficial given the lack of psychotropic effect, compared to agents signalling through CB1 receptors [115]. Moreover, alternative paths of CBD lead to an increase in enzymes involved in protection from oxidative stress and degeneration of dopaminergic neurons, which is characteristic of Parkinson’s disease [114]. THC shows equally expressed activity by reducing the production of IFN-γ and pro-inflammatory IL-12 and increasing the level of anti-inflammatory IL-4 [109]. These results are essential because Parkinson’s disease leads to excessive activation of microglial cells and increases pro-inflammatory cytokines, such as TNF-α, IL-1β, IL-2, and IL-6 [104].

Alzheimer’s disease (AD) constitutes an enormous therapeutic challenge for modern neurology. Progressive neurodegeneration of the CNS results in cognitive disorders at different severity stages. Increased expression of CB2 receptors in brain cells of deceased AD patients has been confirmed [104]. Synthetic agonists of cannabinoid receptors are a promising target for research, as they led to reduced microglial activation, decreased TNF-α release, and lowered the expression of CD40—a protein involved in phagocytosis of microglial cells [104].

Benefits of using cannabinoids are also seen in Huntington’s disease, an autosomal dominant disorder resulting in abnormal structure of the huntingtin protein. The neurodegenerative changes concern primarily cortical neurons and striatum, which is clinically manifested by motor dysfunctions and dementia [116]. A significant reduction of CB1 receptor expression is observed in brain samples of deceased Huntington’s disease patients [117]. Cannabinoids signaling through CB2 receptors seem to have particular therapeutic potential. In mice with experimentally induced excitotoxicity, selective agonist attenuated inflammatory response, reduced cerebral edema and neuron loss in striatum, and improved motor functions [118]. Contrary effects were observed in mice with CB2 receptor deficiency [118].

In vitro and in vivo studies have shown that cannabigerol (CBG) and its synthetic quinone derivative (VCE-003.2) have neuroprotective potential to reduce the severity of neurologic illnesses, such as Huntington’s disease, amyotrophic lateral sclerosis, Parkinson’s disease, and multiple sclerosis, primarily mediated by PPAR-γ [27,119,120,121,122]. CBG and VCE-003.2 have been shown to reduce inflammatory molecules, such as TNF-α, IL-1β, IL-6, prostaglandin E2 (PGE2), and MIP-1α in rat microglial cells treated with lipopolysaccharide (LPS) [27]. Both compounds also reduce glutamate-induced oxidative cell death in mouse hippocampal cells [27]. CBG amounts to less than 10% of the cannabinoid fraction in *Cannabis sativa* L. and therefore its potential has been overlooked for many years [123].

Apart from the neuroprotective and antioxidative properties, there is data providing evidence of mitigating effects of cannabinoids on symptoms of MS, caused by demyelination of neuronal fibers, manifested mainly by spasticity, pain, or tremor [124,125]. Cannabis extracts seem to improve patients’ condition in MS, although the effect is not accompanied by changes in serum inflammatory markers, which suggests the symptom relief is a result of different mechanisms [12]. CBD-dependent IL-17 downregulation and inhibition of T cell proliferation seem to present promising immunomodulating and disease-modifying effects. However, available evidence does not provide information about molecular targets involved in the complex pharmacological profile of CBD and requires further examination [126].

Presented reports are insufficient to suggest the therapeutic benefits of using cannabinoids in patients suffering from neurodegenerative diseases associated with inflammatory processes. Nevertheless, it provides a good rationale for further clinical trials.

## 7. Summary

**Figure 2:**
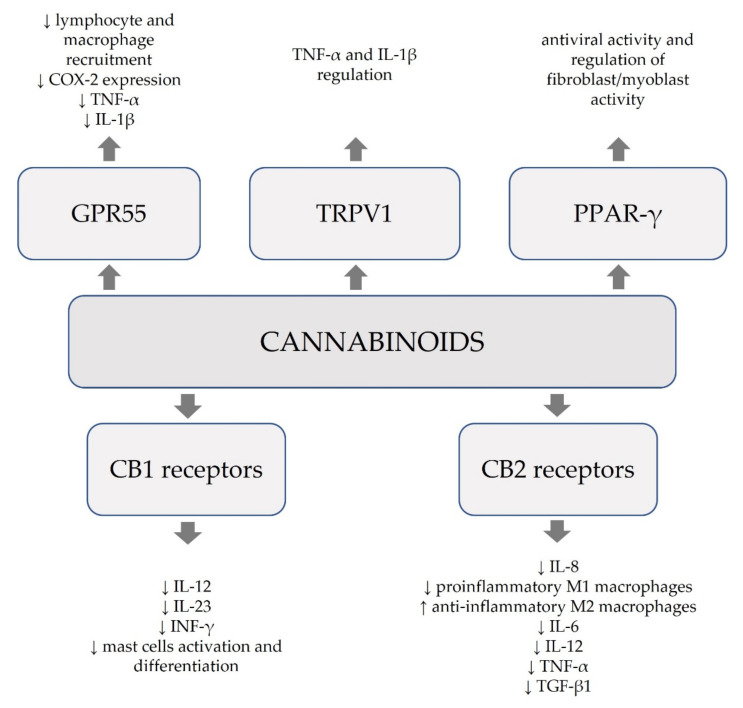
Anti-inflammatory, immunological, and antioxidative modes of action of cannabinoids.

Multifaceted modes of action cannabinoids include their involvement in immunological processes, anti-inflammatory and antioxidative effects (Figure 2). The scarce evidence suggests their potential in clinical use, though their role cannot be determined. There is a need for well-designed clinical trials to determine prospects for the use of cannabinoids in inflammatory conditions.

## Figures and Tables

**Table 1:** Mechanisms of action of anti-inflammatory cannabinoids in preclinical studies.

Type of Cannabinoids	Mode of Action	Effect/Process	Reference
Cannabinoids (generally)	inhibition of leukocyte proliferation, induction of apoptosis of T lymphocytes and macrophages, decrease in the excretion of pro-inflammatory cytokines	mitigation of the inflammatory response dependent on the specific kind of the applied cannabinoid, dosage, the frequency of administration, and the cells they mediate	[17,19]
	increased production of anti-inflammatory eicosanoids	difference between cannabinoids and cyclooxygenase-2 inhibitors that suppress the synthesis of the pro-inflammatory eicosanoids	[5]
	omnidirectional influence of cannabinoids	possible benefits associated with the anti-inflammatory effects, along with the positive response to attempts to relieve other symptoms	[22]
Cannabinoids (natural and synthetic)	modulation of releasing pro-inflammatory mediators (IL-1β, TNF-α, and COX-2) and leucocyte recruitment mediated by G protein-coupled receptor 55 (GPR55)	involvement in development of neuropathic and inflammatory pain	[15,23]
CBD -phytocannabinoid of *Cannabis sativa* L.	affinity to PPAR-γ, 5-HT_1A_, adenosine A_2A_, and TRP	antioxidant, immunomodulatory and anti-inflammatory effects, mitigation of uncontrolled cytokine production, antiviral activity, regulation of fibroblast/myoblast activation, amelioration of lung function	[14,24]
THC- phytocannabinoid of *Cannabis sativa* L.	inhibition of the release of proinflammatory cytokines-IFNγ, TNF-α, IL-17A, and IL-22	possible protective capacity of cannabinoids against carcinogenesis in the colon	[25]
	functions independent of CB1 and CB2 receptors	inhibition of the production of IFN-γ by T lymphocytes and decrease in the release of pro-inflammatory chemokines and cytokines induced by IFN-γ	[26]
CBG- phytocannabinoid of *Cannabis sativa* L.	reduction of the inflammatory molecules -TNF-α, IL-1β, IL-6, PGE2 MIP-1α in microglial cells and glutamate-induced oxidative cell death in hippocampal cells	anti-inflammatory and antioxidative properties, neuroprotective potential to reduce the severity of neurologic illnesses	[27]
ECS	decrease in MHC class II on the surface of dendritic cells	modulation of immunological processes, antigen presentation and inhibition of peripheral T-cell activation	[18]
CB1 receptor agonists	limitation of the activation and differentiation of mast cells	anti-inflammatory effect possibly beneficial in treating chronic inflammatory skin disorders	[28,29]
	inhibition of the release of pro-inflammatory cytokines IL-12, IL-23, and INF-γ by T lymphocytes		[30]
CB2 receptor agonists	stimulation of the receptors expressed in B lymphocytes, NK cells, monocytes, neutrophils, and leucocytes CD8 and CD4	mitigation of inflammatory response, immunomodulatory effects	[17]
	inhibition of the release of IL-8 induced by TNF-α in colon epithelial cells	influence on immunological homeostasis of the intestine	[31]
	significant decrease in pro-inflammatory M1 macrophages, increase in anti-inflammatory M2 macrophages; inhibition of the release of cytokines IL-6, IL-12, CD86, iNOS; decrease in MCP-1, SDF-1, IL-6, IL-1β, TNF-α, TGF-β1, VEGF	reduced infiltration of neutrophils and macrophages, increased keratinocyte proliferation, and faster wound healing	[32]
	decrease in the production of pro-inflammatory cytokines and immune cell proliferation	possible benefits in limiting inflammatory response in SARS-CoV-2 infection	[33]
AJA	preferentially binding to CB2 receptors; inhibition of IL-1β release	potential role in inhibiting tumor progression by reducing inflammation in the microenvironment of the tumor	[34]
non-cannabinoid compounds of cannabis- olivetol, cannflavin, and BCP	mechanisms mediated by CB2 receptors; decrease in the production of pro-inflammatory mediators	anti-inflammatory effects mediated by CB2 receptor-an important therapeutic target in many diseases	[6,7]

5-HT_1A_, serotonin 1A receptor; AJA, ajulemic acid; BCP, beta-caryophyllene; CB1, cannabinoid receptor 1; CB2, cannabinoid receptor 2; CBD, cannabidiol; CBG, Cannabigerol; COX-2, cyclooxygenase-2; ESC, endocannabinoid system; GPR55, G protein-coupled receptor 55; IFN-γ, interferon γ; IL, interleukin; iNOS, inducible nitric oxide synthase; MCP-1, monocyte chemoattractant protein-1; MHC, major histocompatibility complex; MIP-1α, macrophage inflammatory protein; PGE2, prostaglandin E2; PPAR-γ, peroxisome proliferator-activated receptors-gamma γ; SDF-1, stromal cell-derived factor 1; TGF-β1, transforming growth factor-beta 1; THC, tetrahydrocannabidiol; TNF-α, tumor necrosis factor α; TRP, transient receptor potential; VEGF, vascular endothelial growth factor.

**Table 2:** Clinical effects of action in the gastrointestinal tract.

Study	Study Design	Number of Participants	Drug/Substance	Dosage	Condition	Treatment Duration	Endpoints/ Measures	Outcomes	Limitations
Lal 2011 [44]	questionnaire based survey	UC 100; CD 191	self-administration of cannabis		UC/CD		quality of life improvement	symptom relief and improved quality of life	patient-reported survery; high bias
Kafil 2018 [41]	randomized controlled trial	21	cannabis cigarettes containing THC	115 mg	active CD	eight weeks	clinical response and remission	positive response to the treatment and clinical remission, mild side effects	high bias
Kafil 2018 [41]	randomized controlled trial	50	cannabis oil consisting of 15% CBD and 4% THC		active CD	eight weeks	improvement in mean quality of life score and CDAI score	no clinical remission, improved quality of life, lowered CDAI score	low certainty evidence
Storr 2014 [45]	questionnaire based survey	313	self-administration of cannabis		IBD			relief of abdominal pain, abdominal contractions, joint pain and diarrhea; higher risk of surgery in patients with Crohn’s disease	patient-reported study; high bias
Lahat 2012 [46]	questionnaire based survey	13	cannabis		IBD	three months	measurement of quality of life, disease activity and weight gain	improved quality of life, clinical disease activity and increased body mass index	high bias

CBD—cannabidiol; CD—Crohn’s disease; CDAI—Crohn’s Disease Activity Index; IBD—Inflammatory bowel disease; THC—tetrahydrocannabinol; UC—ulcerative colitis.

## Data Availability

No new data were created or analyzed in this study. Data sharing is not applicable to this article.
